# Evaluation in gene polymorphic α-ACTN3 at blumenau in individuals for better performance in sport

**DOI:** 10.1186/1753-6561-8-S4-P16

**Published:** 2014-10-01

**Authors:** Ana Kelly Pitlovanciv, Laura Cristine Branco, Vanessa Rosália Remualdo

**Affiliations:** 1 LaboratórioGenolab, Blumenau, SC, 89.010-325, Brazil

## Background

3 α-actin (α-ACTN3) isoform is characteristic of fast fibers, expressed only in type II fibers, which plays structural and regulatory functions in cytoskeletal organization and muscle contraction. Stabilizes sarcomeres during fast and forceful contraction of muscle fibers used during sports activities that require explosion, such as race. Gene polymorphism was identified in the α-called ACTN3 R577X results in exchange of C to T at nucleotide position 1747 of exon 16, that is, a mutation resulting in the conversion of arginine (R allele) by premature termination codon (allele X) at amino acid 577. This change leads to lack expression of α-ACTN3 in individuals homozygous for the allele X, which supposedly causes decrease in muscle mass and affect the performance activities that require muscle contractions with high levels of strength and / or speed. The deficiency of α-ACTN3 does not result in a pathological phenotype as muscular dystrophy or myopathy. We analyzed the frequency and estimated genotype α-ACTN3 gene in individuals from Blumenau to guide them in the perfomance physical activity.

## Methods

For analysis, was used for DNA extraction blood sample by phenol-chloroform method, followed by PCR and Real-Time results analyzed by the graph generated. Protocol was used reagents as recommended by Applied Biosystems ^®^.

## Results and conclusions

We obtained the number of 23 subjects, 12 females and 11 males, aged 22-60 years. The distribution of genotypes was 39.1% for genotype RR, RX, and 47% to 13% to XX. The result for females was 50% for RX, followed by 33.3% to 16.7% for RR and XX. For males was obtained a number of 45.5% for RR, 45.5% for RX and a small number for XX 9.1%. The result of the polymorphism has examined the possibility of directing sports practices specific to individuals according to their genetic sensitivity training. For individuals that express α-ACTN3 gene (genotype RR or RX) may have advantage in ways that require explosion and muscle strength when compared with individuals with XX genotype.
